# THE COVID-19 EFFECT: ADAPTING FAMILY CARE TO A PANDEMIC CONTEXT

**DOI:** 10.1093/geroni/igac059.472

**Published:** 2022-12-20

**Authors:** Amanda Leggett, Laura Gitlin

## Abstract

The COVID-19 pandemic carries risk for severe complications and mortality among older adults, placing their family caregivers in key support roles in helping their care recipients stay safe, maintain function, and abide by preventative recommendations. Yet such preventative safety precautions, changes to social support structures, and impacts on healthcare access may pose challenges with which caregivers must cope. This symposium considers how family caregivers adapted their care practices and adjusted to care-related challenges during the COVID-19 pandemic. First, Dr. Amanda Leggett presents data on pandemic-specific care-related challenges and supports experienced by dementia caregivers and their association with well-being and stress process outcomes. Ms. Sara Masoud shares mixed-methods data from persons living with dementia, caregivers, and healthcare professionals on their health and quality of life during the pandemic. Mr. Jiaming Liang presents dyadic findings from the National Study of Caregiving on persons living with dementia and their caregivers’ perceptions of COVID-19 and personal and social COVID-specific preventative behaviors. Finally, Dr. Sheria Robinson-Lane offers a diverse caregiving perspective by presenting dyadic qualitative data on COVID-19 patients who were intubated in the hospital and their family caregiver, and offering themes on how caregivers adjusted to their new care role. To conclude, our discussant Dr. Laura Gitlin will offer insight on cross-cutting implications across studies and offer perspective on how lessons learned through pandemic caregiving may inform the field more broadly and enhance caregiver well-being beyond pandemic contexts.

